# Downregulation of SFRP5 expression and its inverse correlation with those of MMP-7 and MT1-MMP in gastric cancer

**DOI:** 10.1186/1471-2407-9-224

**Published:** 2009-07-09

**Authors:** Chenghai Zhao, Xianmin Bu, Ning Zhang, Wei Wang

**Affiliations:** 1 Department of Pathophysiology, China Medical University, Shenyang, PR China; 2 Department of General Surgery, Shengjing Hospital, Shenyang, PR China

## Abstract

**Background:**

As negative regulators in Wnt signaling, Secreted Frizzled-Related Proteins (SFRPs) are downregulated in a series of human cancers; and specifically, some matrix metalloproteinases (MMPs), including MMP-2, MMP-7, MMP-9 and MT1-MMP, are frequently overexpressed in gastric cancer. The aim of this study is to determine the expression status of SFRP5 in gastric cancer and explore the correlation between both the expression of SFRP5 and that of these MMPs in this cancer.

**Methods:**

Expression of SFRP5, MMP-2, MMP-7, MMP-9 and MT1-MMP was determined by real-time PCR, RT-PCR or Western blotting. The methylation status of *SFRP5 *was detected by Methylation-specific PCR (MSP). Cell lines with *SFRP5 *methylation were demethylated by a DNA methyltransferase inhibitor 5-Aza-2'-deoxycytidine (DAC). KatoIII cells were transfected with pcDNA3.1 *SFRP5 *vector to strengthen SFRP5 expression. To abrogate SFRP5 expression in MKN1 cells, *SFRP5 *RNAi plamid was used to transfect them.

**Results:**

SFRP5 expression was remarkably downregulated in 24 of 32 primary gastric cancer specimens, and even was not detectable in 5 of 8 gastric cancer cell lines. MMP-7 and MT1-MMP mRNA showed a stronger expression in these 24 specimens compared to the other 8 specimens. They also showed higher levels in gastric cancer cell lines AGS and NCI-N87 which had no SFRP5 expression, compared to MKN1 with strong SFRP5 expression. However, they were significantly downregulated, with SFRP5 expression restored in AGS and NCI-N87; and were considerably upregulated with it abrogated in MKN1.

**Conclusion:**

The results indicate there are frequent occurrences of downregualtion of SFRP5 expression in gastric cancer, primarily due to *SFRP5 *methylation. It seems to be responsible for the upregulation of MMP-7 expression and MT1-MMP expression on the ground that they are inversely correlated with SFRP5 expression.

## Background

All the five secreted glycoproteins in Secreted Frizzled-Related Protein (SFRP) family contain a cysteine-rich domain (CRD) homologous to that of Wnt receptor Frizzled proteins. It is noted that via the CRD, they compete with the Frizzled proteins for Wnt binding, interfering with Wnt signaling. Downregulation of them can lead to aberrant activation of the Wnt pathway and induce tumorigenesis. SFRP1 is particularly well known for its frequent downregulation in human tumors, mainly due to hypermethylation of *SFRP1 *promoter [1-3]. Other members of this family, such as SFRP2, SFRP4 and SFRP5 [4,5], were also found to exert their roles in human cancers. These studies have labeled SFRPs as the candidate tumor suppressors in carcinogenesis.

It is also noted that matrix metalloproteinases (MMPs) are involved in a variety of physiological and pathological conditions, among which, their link with cancer has been most extensively studied. As a family of zinc-dependent endopeptidases, MMPs participate in the degradation of extracellular matrix components. In addition, their specific substrates also include latent growth factors, growth factor binding proteins, chemokines and cell adhesion molecules. Naturally, MMPs are known not only for their roles in invasion and metastasis of tumor, but also in growth and angiogenesis of it. Overexpression of MMPs has been observed in a series of cancers, with that of MMP-2[6,7], MMP-7[8,9], MMP-9[10] and MT1-MMP[11], in particular, typically present in gastric cancer.

Our initial finding showed that SFRP5 expression is significantly downregulated in gastric cancer, while the subsequent quantitative real-time PCR analysis revealed that MMP-2, MMP-7, MMP-9 and MT1-MMP are all upregulated in it. Such a finding that these MMPs are the targets of Wnt signaling [12-15] led to our initial hypothesis: there may be a link between downregulation of SFRP5 and overexpression of these MMPs in gastric cancer. Another hypothesis was that the upregulation of these MMPs may be related to aberrant activation of Wnt signaling pathway due to SFRP5 downregulation.

## Methods

### Cell lines and tissue specimens

The human gastric cancer cell lines AGS, NCI-N87 and KatoIII were obtained from the American Type Culture Collection (Rockville, MD, USA); and MKN1, MKN28, MKN45, SGC-7901 and HGC-27 were obtained from Keygen Biotech. Co. (Nanjing, China). These cell lines were cultured in RPMI 1640 supplemented with 10% fetal bovine serum, at 37°C in a humid incubator with 5% CO_2_. 32 primary gastric cancer specimens and their corresponding matched cancer adjacent normal tissue specimens were obtained from patients under operation at Shengjing hospital, Chinese Medical University. Informed consent was obtained from all patients before collection of the specimens, which were frozen in liquid nitrogen immediately after surgical removal. Haematoxylin- and eosin-staining sections were prepared for assessment of the percentage of tumor cells, and only specimens with >70% tumor cells were selected for analysis. This study was carried out with the approval of the ethical committee of China Medical University.

### RT-PCR and real-time reverse-transcription PCR

Total RNA was isolated from tissues and cell lines by Trizol (Takara, Dalian, China) according to the protocol supplied by the manufacturers. cDNA was synthesized from 1 μg RNA using random 9 primer and AMV reverse transcriptase. RT-PCR was performed using RNA PCR 3.0 Kit (Takara, Dalian, China). Real-time PCR was performed using the LightCycler system together with the LightCycler DNA Master SYBR Green I Kit (LightCycler, Roche Diagnostics). The housekeeping gene glyceraldehyde-3-phosphate dehydrogenase (*GAPDH*) was used as an internal control. Gene expression was quantified by the comparative CT method, normalizing CT values to *GAPDH *and calculating relative expression values. Primer sequences for *SFRP5*, *MMP-2*, *MMP-7*, *MMP-9*, *MT1-MMP *and *GAPDH *are shown in table 1.

**Table 1:** Primer sequences for RT-PCR

Gene		Sequence	Product size	Annealling temperature
MMP-2	Sense	5'-GGATGATGCCTTTGCTCG-3'	487 bp	55°C
	Anti-sense	5'-ATAGGATGTGCCCTGGAA-3'		
MMP-7	Sense	5'-CTTCAGGCAGAACATCCA-3'	220 bp	49°C
	Anti-sense	5'-ATTTATTGACATCTACCCAC-3'		
MMP-9	Sense	5'-AGGACGGCAATGCTGATC-3'	127 bp	58°C
	Anti-sense	5'-TCGTAGTTGGCGGTGGTG-3'		
MT1-MMP	Sense	5'-CCTGCGTCCATCAACACT-3'	481 bp	57°C
	Anti-sense	5'-TCACCTCCGTCTCCTCCTC-3'		
SFRP5	Sense	5'-AGGGTAAGGGAAAGGTGGAG-3'	379 bp	60°C
	Anti-sense	5'-GAGAAGCAGAGGCTGAGGAA-3'		
GAPDH	Sense	5'-GGGAAACTGTGGCGTGAT-3'	309 bp	56°C
	Anti-sense	5'-AAAGGTGGAGGAGTGGGT-3'		

### Methylation-specific PCR and DNA demethylation

Genomic DNA was extracted from tissues and cell lines by a standard phenol/chloroform extraction and ethanol precipitation procedure. Methylation of *SFRP5 *was detected by Genmed MSP Kit (Genmed, Shanghai, China). The procedure was performed according to the manufacturer's instructions. Primers for *SFRP5 *methylated sequence and unmethylated sequence were described in reference [4]. Cell lines with *SFRP5 *methylation were demethylated by 5-Aza-2'-deoxycytidine (DAC) (2 μM, Sigma, St Louis, MO, USA). Cells were seeded at a density of 3 × 10^4 ^cells/cm^2 ^in a 24 well plate on day 0, and exposed to DAC on day 1, 2, and 3. After each treatment, the cells were cultured in fresh medium. Control cells were incubated without the addition of DAC. Cells were harvested on day 4 for experiment.

### Western blotting

Standard protocol was used. In brief, a total of 10 μg protein of each sample was run on a 12% sodium dodecyl sulfate (SDS)/acrylamide gel. The proteins on acrylamide gel were transferred to a nylon membrane, which was blocked overnight (4°C in PBS with 0.1% Tween and 10% milk powder). Polyclonal antibodies for SFRP5 (Santa Cruz, CA, America), MMP-2, MMP-7, MMP-9 and MT1-MMP (Abcam, Cambridge, United Kingdom), and the corresponding secondary antibodies (Santa Cruz, CA, America) were applied before immunoblotting. The human gene *β-actin *(Santa Cruz, CA, America) was used as an internal control.

### RNA interference

*SFRP5 *RNAi plasmid and nonsilencing control RNAi plasmid were purchased from Takala Biotech. Co. (Dalian, China). The target sequence of *SFRP5 *siRNA was 5'-AAGAAGAATAAGGAGATGAAGTT-3', corresponding to 1022 to 1044 of the human *SFRP5 *cDNA sequence (GenBank: NM 003015). Cells were seeded into a 24-well plate at a density of 2 × 10^5^. On the following day cells were transfected with *SFRP5 *siRNA or Control siRNA using Lipofectamine 2000 (Invitrogen, Paisley, United Kingdom) according to the manufacturer's instructions.

### Construction of expression plasmids and transient transfection

The full-length pcDNA3.1 (Invitrogen, Paisley, United Kingdom) *SFRP5 *vector was made by cloning of the full-length PCR product of *SFRP5 *with PFU DNA polymerase (Invitrogen, Paisley, United Kingdom). All the plasmid sequences were confirmed by DNA sequencing. For transient transfection experiments, cells were plated in a 24-well plate at a density of 2 × 10^5 ^24 hours before transfection. Lipofectamine 2000 (Invitrogen, Paisley, United Kingdom) was used to perform transfection with 2.0 μg pcDNA3.1 *SFRP5 *vector or 2.0 μg pcDNA3.1 empty vector (as control) according to the manufacturer's protocol.

### Statistical analysis

χ^2 ^test was used to compare the methylation frequency between tumors and tumor adajcent normal tissues. The correlation between methylation and mRNA expression in primary gastric cancer was analyzed by Fisher's exact test. Mann-Whitney *U*-test was used to compare mRNA expression between tumors and tumor adjcent normal tissues. mRNA expression in gastric cell lines was compared using Student's *t*-test or one way ANOVA. Statistical analysis was carried out using SPSS version 13.0 (SPSS, Chicago, IL, USA). Difference was considered significant when *P*-value was < 0.05.

## Results

### Downregulation of SFRP5 expression in primary gastric cancer specimens

Expression of SFRP5 mRNA was determined by real-time reverse-transcription PCR in 32 gastric cancer specimens and corresponding matched normal tissue specimens. *SFRP5 *expression in each specimen was standardized to *GAPDH *expression. As an attempt to compare the expression difference between tumors and normal tissues, we normalized *SFRP5 *expression in tumors to the mean of *SFRP5 *expression in matched normal group. The results showed SFRP5 mRNA expression was downregulated by more than 10-fold in 24 of 32 (75%) tumors compared to normal tissues. In 4 of 32 (12.5%)tumors, there was a less than 10-fold downregulation in expression level, and *SFRP5 *expression was up-regulated in 4 of 32 (12.5%) tumors (Table 2).

**Table 2:** SFRP5 expression and SFRP5 methylation in primary gastric cancer specimens.

No.	Sex	Age	SFRP5 expression	SFRP5 methylation	
				
				Cancer	Normal
1	M	76	6 × 10^-4^	M	U
2	M	65	8 × 10^-4^	M	M
3	M	65	4 × 10^-3^	M	U
4	F	67	4 × 10^-3^	M	U
5	F	54	4 × 10^-3^	M	U
6	M	81	6 × 10^-3^	M	U
7	M	59	6 × 10^-3^	M	U
8	M	47	7 × 10^-3^	M	M
9	M	76	8 × 10^-3^	M	U
10	F	68	9 × 10^-3^	U	U
11	M	72	9 × 10^-3^	M	U
12	F	76	9 × 10^-3^	M	U
13	M	59	2 × 10^-2^	M	M
14	M	77	2 × 10^-2^	M	U
15	M	59	3 × 10^-2^	M	U
16	F	70	4 × 10^-2^	U	U
17	M	58	4 × 10^-2^	M	U
18	M	64	4 × 10^-2^	M	U
19	M	82	5 × 10^-2^	M	U
20	F	49	6 × 10^-2^	M	U
21	M	59	7 × 10^-2^	M	M
22	F	44	8 × 10^-2^	M	U
23	M	65	8 × 10^-2^	M	U
24	M	74	9 × 10^-2^	U	U
25	F	72	3 × 10^-1^	U	U
26	F	60	5 × 10^-1^	U	U
27	M	57	6 × 10^-1^	U	U
28	M	71	6 × 10^-1^	U	U
29	M	69	1.2	M	M
30	M	68	1.4	U	U
31	M	78	2.6	U	U
32	M	66	5.7	U	U

**Figure 1:**
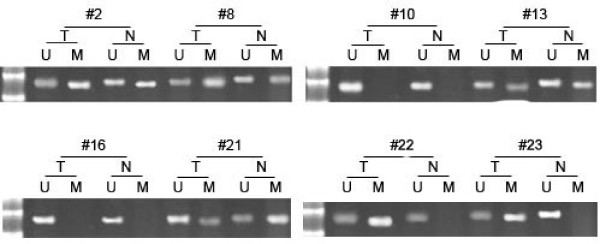
**Analysis of SFRP5 methylation in primary gastric cancer specimens (T) and matched normal tissue specimens (N) by Methylation-specific PCR**. Shown are representive results from specimen #2, #8, #10, #13, #16, #21, #24 and #29. M: methylated primers; U: unmethylated primers.

Next we performed methylation-specific PCR to detect the methylation status of *SFRP5 *in the same cohort of tumors and matched normal tissues. The detection reported *SFRP5 *methylation in 22 (69%) of the 32 gastric cancer specimens. In contrast, in 32 matched normal tissues, only 5 (16%) was reported with *SFRP5 *methylation (χ^2 ^= 18.515, *P *< 0.001)(Figure 1). To further investigate the role of promoter methylation in the downregulation of SFRP5 expression in gastric cancer, we defined SFRP5 mRNA downregulation more than 10-fold as negative expression, and SFRP5 mRNA downregulation less than 10-fold or SFRP5 mRNA upregulation as positive expression. The subsequent Fisher's exact test analysis showed *SFRP5 *expression was correlated inversely with *SFRP5 *methylation (21/3 versus 1/7; *P *< 0.001).

### Differential SFRP5 expression in gastric cancer cell lines

**Figure 2:**
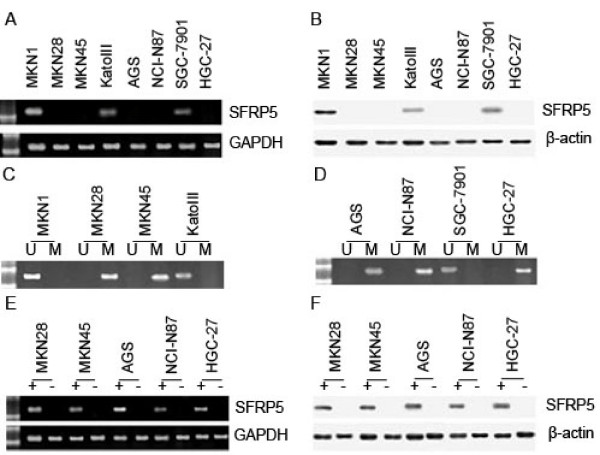
**SFRP5 expression and SFRP5 methylation in gastric cancer cell lines**. (A) SFRP5 mRNA was not detected in MKN28, MKN45, AGS, NCI-N87 and HGC-27 by RT-PCR. (B) SFRP5 protein was not detected in MKN28, MKN45, AGS, NCI-N87 and HGC-27 by Western blotting. (C) SFRP5 methylation was found in MKN28, MKN45, AGS, NCI-N87 and HGC-27 by methylation-specific PCR. (D) SFRP5 mRNA re-expression was found in MKN28, MKN45, AGS, NCI-N87 and HGC-27 after treatment with DAC by RT-PCR. (E) SFRP5 protein re-expression was found in MKN28, MKN45, AGS, NCI-N87 and HGC-27 after treatment with DAC by Western blotting. M: methylated primers; U: unmethylated primers.

RT-PCR and Western blotting were used to examine SFRP5 expression in gastric cell lines MKN1, MKN28, MKN45, KatoIII, AGS, NCI-N87, SGC-7901 and HGC-27, and the examination showed SFRP5 expression varied in these cell lines: a strong expression of it in MKN1, a weak but detectable one in KatoIII and SGC-7901, and none in MKN28, MKN45, AGS, NCI-N87 and HGC-27 (Figure 2A, B).

To investigate whether SFRP5 expression was correlated with *SFRP5 *methylation, methylation-specific PCR was used to detect the *SFRP5 *methyaltion status in these gastric cell lines. The detection revealed that *SFRP5 *was methylated in all 5 cell lines without SFRP5 expression (MKN28, MKN45, AGS, NCI-N87 and HGC-27), and in contrast, *SFRP5 *methyaltion was not found in MKN1, KatoIII and SGC-7901 (Figure 2C, D). Next, with a DNA methyltransferase inhibitor 5-Aza-2'-deoxycytidine (DAC), we treated MKN28, MKN45, AGS, NCI-N87 and HGC-27, where *SFRP5 *was methylated and silenced, and interestingly, both SFRP5 mRNA and SFRP5 protein were restored in all these 5 cell lines (Figure 2E, F).

### Upregulation of expression of some MMPs in primary gastric cancer specimens

**Figure 3:**
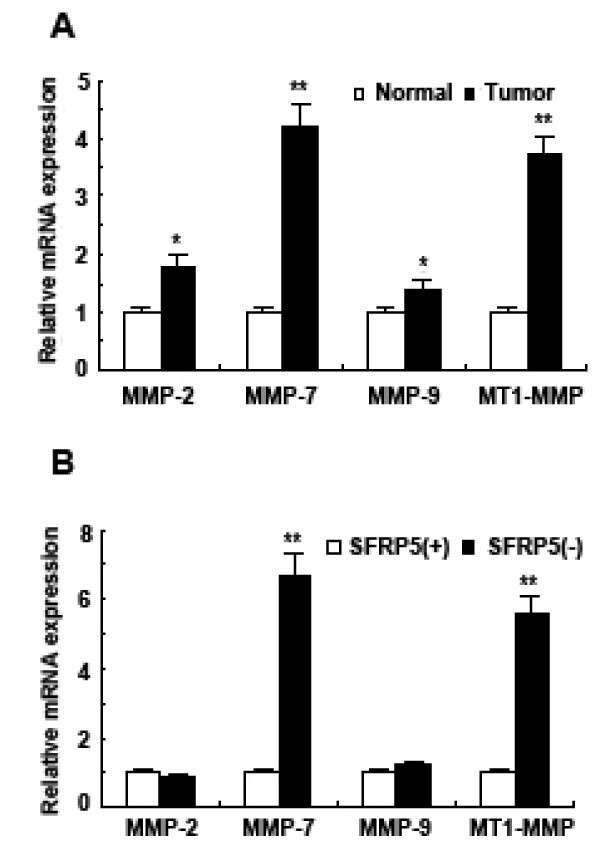
**The mRNA expression of MMP-2, MMP-7, MMP-9 and MT1-MMP in primary gastric cancer specimens by Real-time PCR**. (A) Gastric cancer specimen (T) expressed higher levels of MMP-2, MMP-7, MMP-9 and MT1-MMP compared to matched normal tissue specimens (N). Data are expressed as mean ± SD, n = 32, *P < 0.01, **P < 0.001, *vs *Normal. (B) SFRP5 expression-negative gastric cancer specimens (SFRP5-) expressed higher levels of MMP-7 and MT1-MMP compared to SFRP5 expression-positive (SFRP5+) gastric cancer specimens. Data are expressed as mean ± SD, n = 24 and 8, respectively, **P < 0.001, *vs *SFRP5(+).

Real-time reverse-transcription PCR was performed to determine the mRNA expression of some members in MMP family, including MMP-2, MMP-7, MMP-9 and MT1-MMP in 32 gastric cancer specimens and corresponding matched normal tissue specimens. Furthermore, Mann-Whitney *U*-test was used to analyze the difference of mRNA expression between these two groups. The results showed that *MMP-2*, *MMP-7*, *MMP-9 *and *MT1-MMP *were all significantly upregulated in gastric cancer specimens compared to matched normal tissue specimens (For both *MMP-2 *and *MMP-9*, *P *< 0.01; for both *MMP-7 *and *MT1-MMP*, *P *< 0.001) (Figure 3A).

As for the correlation between expression of SFRP5 and those of these MMPs, mRNA expression of these MMPs was examined when we compared gastric cancer specimens with negative SFRP5 expression to those with positive one. The study showed levels of MMP-7 mRNA and MT1-MMP mRNA were significantly higher in the former group than those in the latter group (*P *< 0.001, respectively). However, such a difference was not found in *MMP-2 *and *MMP-9 *(Figure 3B).

### Differential expression of some MMPs in gastric cancer cell lines

**Figure 4:**
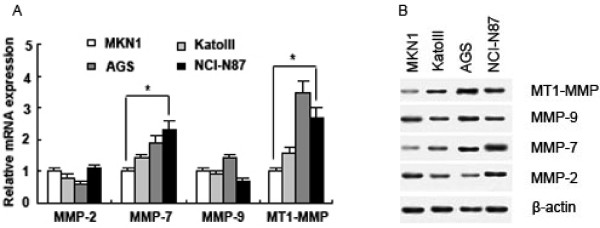
**The expression of MMP-2, MMP-7, MMP-9 and MT1-MMP in gastric cancer cell lines MKN1, KatoIII, AGS and NCI-N87**. (A) Differential mRNA expression of MMP-2, MMP-7, MMP-9 and MT1-MMP in MKN1, KatoIII, AGS and NCI-N87 was found by real-time PCR. Data are expressed as mean ± SD, n = 3, *P < 0.05. (B) Differential protein expression of MMP-2, MMP-7, MMP-9 and MT1-MMP in MKN1, KatoIII, AGS and NCI-N87 was found by Western blotting.

We performed real-time reverse-transcription PCR and Western blotting to determine the expression of MMP-2, MMP-7, MMP-9 and MT1-MMP in gastric cell lines MKN1 (with strong SFRP1 expression), KatoIII (with weak SFRP1 expression), AGS and NCI-N87 (without SFRP1 expression). The results revealed that expression of these MMPs varied in these cell lines (Figure 4A, B), and one way ANOVA analysis showed both MMP-7 mRNA expression and MT1-MMP mRNA expression were correlated inversely with SFRP5 expression in these cell lines (*P *< 0.001, respectively). However, no significant correlation was found between SFRP5 expression and MMP-2 expression or MMP-9 expression (Figure 4A).

### Correlation between SFRP5 expression and MMP expression

**Figure 5:**
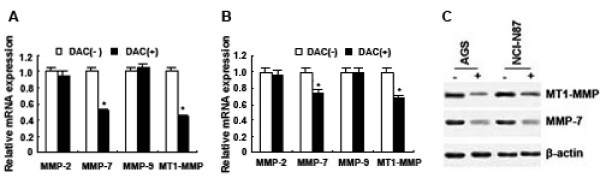
**The expression of MMP-2, MMP-7, MMP-9 and MT1-MMP in gastric cancer cell lines AGS and NCI-N87 after treatment with DAC**. (A) Real-time PCR analysis showed MMP-7 mRNA and MT1-MMP mRNA were downregulated in AGS after treatment with DAC. Data are expressed as mean ± SD, n = 3, *P < 0.001, *vs *DAC(-). (B) Real-time PCR analysis showed MMP-7 mRNA and MT1-MMP mRNA were downregulated in NCI-N87 after treatment with DAC. Data are expressed as mean ± SD, n = 3, *P < 0.001, *vs *DAC(-). (C) Weatern blotting analysis showed MMP-7 protein and MT1-MMP protein were downregulated in AGS and NCI-N87 after treatment with DAC.

To further explore the correlation between SFRP5 expression and MMP expression, first DAC was employed to treat cell lines AGS and NCI-N87, where *SFRP5 *was methylated. The results showed that SFRP5 mRNA and SFRP5 protein were re-expressed in these two cells after the treatment (Figure 2E, F). Then we examined the expression of MMP-2, MMP-7, MMP-9 and MT1-MMP in these two cell lines by real-time PCR and Western blotting (Figure 5A, B, C); and the examination revealed that MMP-7 mRNA and MT1-MMP mRNA in DAC-treated AGS were downregulated compared to DAC-untreated AGS (*P *< 0.001, respectively) (Figure 5A). Similar results were also found in cell line NCI-N87 (*P *< 0.001, respectively) (Figure 5B).

**Figure 6:**
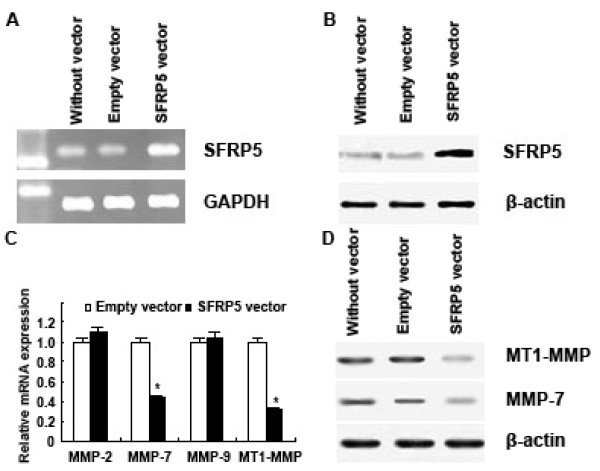
**Transfecting katoIII with pcDNA3.1 SFRP5 vector**. (A) Increased expression of SFRP5 mRNA was detected in katoIII after SFRP5 transfection treatment by RT-PCR. (B) Increased expression of SFRP5 protein was detected after SFRP5 transfection treatment by Western blotting. (C) Real-time PCR analysis showed MMP-7 mRNA and MT1-MMP mRNA were downregulated in katoIII after SFRP5 transfection treatment. Data are expressed as mean ± SD, n = 3, *P < 0.001, *vs *Empty vector. (D) Weatern blotting analysis showed MMP-7 protein and MT1-MMP protein were downregulated in katoIII after SFRP5 transfection treatment.

Next, pcDNA3.1 *SFRP5 *vector was employed when katoIII (with a low level expression of SFRP5) was transfected, and pcDNA3.1 empty vector was employed in the counterpart as control. The results showed that SFRP5 expression was elevated in KatoIII after the transfection in the former case (Figure 6A, B). Subsequently the expression of MMP-2, MMP-7, MMP-9 and MT1-MMP in KatoIII was detected by real-time PCR and Western blotting (Figure 6C, D). It is noted in the detection that, after transfection with pcDNA3.1 *SFRP5 *vector, both MMP-7 mRNA and MT1-MMP mRNA were downregulated in KatoIII, compared to control (*P *< 0.001, respectively) (Figure 6C).

**Figure 7:**
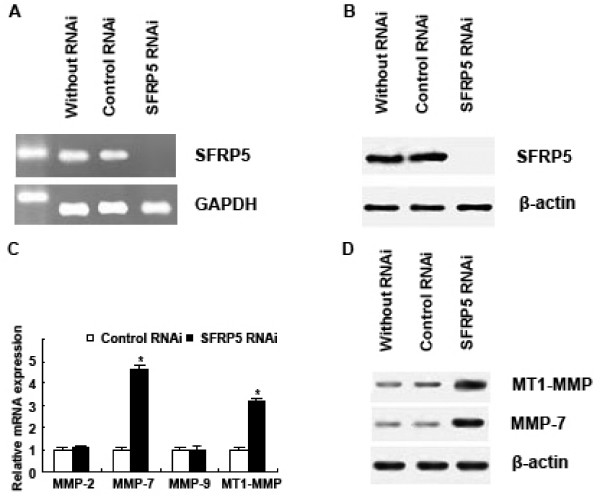
**Silencing SFRP5 by RNAi in MKN1 cells**. (A) SFRP5 mRNA was not detected in MKN1 after SFRP5 RNAi treatment by RT-PCR. (B) SFRP5 protein was not detected in MKN1 after SFRP5 RNAi treatment by Western blotting. (C) Real-time PCR analysis showed MMP-7 mRNA and MT1-MMP mRNA were upregulated in MKN1 after transfection with SFRP5 RNAi plamid. Data are expressed as mean ± SD, n = 3, *P < 0.001, *vs *Control RNAi. (D) Weatern blotting analysis showed MMP-7 protein and MT1-MMP protein were upregulated in MKN1 after transfection with SFRP5 RNAi plamid.

Finally, *SFRP5 *RNAi plamid was used to transfect MKN1 (with strong SFRP5 expression), and correspondingly nonsilencing RNAi plamid was employed in the counterpart as control. It was observed that expression of SFRP5 was absent in MKN1 after transfection with *SFRP5 *RNAi plasmid (Figure 7A, B). Then expression of MMP-2, MMP-7, MMP-9 and MT1-MMP in MKN1 was detected by real-time PCR and Western blotting (Figure 7C, D), and it was noted in the detection that, after transfection with *SFRP5 *RNAi plamid, MMP-7 mRNA and MT1-MMP mRNA were upregulated in MKN1, compared to control (*P *< 0.001, respectively) (Figure 7C).

## Discussion

Lots of studies have shown that aberrant Wnt signaling pathway is involved in carcinogenesis, in fact, alterations of downstream components in Wnt pathway have been found in a series of human cancers. A case in point is the association of mutations of *APC *with most of human colon tumors [16]. In addition, *β-catenin*, another downstream component in Wnt pathway, is also frequently mutated in intestinal cancers [16]. These mutations are known to have led to the decreased degradation of β-catenin before it is translocated and accumulated in the nucleus. There, β-catenin interacts with T-cell factor/lymphocyte enhancer factor (TCF/LEF) to promote the transcription of target genes in Wnt pathway. However, unlike colon cancer, mutations in *APC *and *β-catenin *are quite rare in gastric cancer, despite the occassional β-catenin accumulation in it [17].

Interestingly, within Wnt pathway, the role of alterations of upstream components in gastric carcinogenesis has been drawing attention. The initial focus is the obvious overexpression in certain members of Wnt family, such as Wnt5a[18], Wnt8b[19], Wnt10a[20], and Wnt receptor Frizzled 7[21]. Another spotlight is the hypermethylation and downregulation of Wnt inhibitor SFRP1 in our recent group report[22]. In addition, diminished expression of SFRP2[23] and DKKs[24] (another Wnt inhibitor family) was also observed. Both overexpression of Wnts or their receptor Frizzled proteins, and downregulation of Wnt inhibitors will lead to aberrant activation of Wnt pathway and increase the transcription of Wnt target genes, such as *c-myc*[25], *cyclin *D1[26] and some *MMPs*, which are involved in initiation and progression of tumor. The focus is then shifted onto the significant downregulation (>10-fold) of SFRP5, another member of SFRP family, in 75% of the total gastric cancer specimens, and no detectable expression of it in 5 out of the total 8 gastric cell lines tested. MSP analysis indicated a connection between the absence of SFRP5 and its methylation in all these five cell lines and 21 out of the 24 SFRP5-downregulated gastric cancer specimens, for there is a resumption of it with these cell lines demethylated by DAC. Taken together, these results suggest a strong link between the common SFRP5 downregulation and its methylation in gastric cancer.

The assumption of a link between downregulation of SFRP5 and upregulation of MMPS in gastric cancer is based on the following findings. First, as a putative tumor suppressor gene, SFRP5 is downregulated in a series of human cancers, including gastric cancer; and in most cases, it can lead to aberrant activation of Wnt signal pathway. One recent study reported that *cyclinD1 *and *c-myc*, both known as Wnt targer genes, were significantly downregulated, when the expression of SFRP5 was restored by demethylation agent DAC after it was methylated in breast cancer cell lines. Second, some MMPs, such as MMP-2, MMP-7, MMP-9 and MT1-MMP, are frequenly overexpressed in gastric cancers, primarily in both tumor cells and stromal cells (except MMP-7, which is only expressed in tumor cells). And levels of MMP-2 and MMP-9 are also found elevated in plasma of gastric cancer patients. Thus it is obvious that the overexpression of these MMPs plays important roles in initiation and progression of gastric cancer. Third, other studies have shown Wnt signals can upregulate many members of MMPs family directly or indirectly, including MMP-1[27], MMP-2[12,13], MMP-3[28], MMP-7[14], MMP-9[12,13], MMP-13[13], MMP-26[29], MT1-MMP[15] and MT3-MMP[30].

In this study, real-time PCR was used to determine mRNA levels of *MMP-2*, *MMP-7*, *MMP-9 *and *MT1-MMP *in gastric cancers. We found these four MMPs were all upregulated (from 1.4 folds to 4.2 folds). Next we analyzed the correlation between downregualtion of SFRP5 and upregulation of these four MMPs. The first line of evidence comes from the expression status of SFRP5 in primary gastric cancers. Levels of MMP-7 and MT1-MMP were found significantly high in the SFRP5-negative group. Second, we observed that cell lines AGS and NCI-N87, with no SFRP5 expression due to promoter hypermethyaltion, presented high levels of MMP-7 and MT1-MMP; while cell line MKN1 with strong expression of SFRP5 presented relatively low levels of them. Third, when we restored SFRP5 expression with demethylation agent DAC in AGS and NCI-N87, or enhanced SFRP5 expression with SFRP5 transfection in KatoIII, both MMP-7 expression and MT1-MMP expression are found to have reduced significantly in these cell lines. Finally, to further investigate the action of SFRP5 downregulation on expression of MMPs, we abrogated SFRP5 expression in MKN1 with SFRP5 RNAi. Just as presumed, both MMP-7 expression and MT1-MMP expression were significantly elevated. Taken together, all the lines of evidence suggest a prevalence of SFRP5 downregulation in gastric cancer, which is responsible for the upregulation of the expression of MMP-7 and MT1-MMP.

The mechanism involved in the upregulation of MMP-7 and MT1-MMP by SFRP5 downregulation may be presumably related to aberrant activation of Wnt signals. Now it is well accepted that aberrant activation of Wnt signaling pathway may induce carcinogenesis, and that these Wnt-related tumors often have increased invasiveness and metastasis, which is linked to some members in MMP family. Studies have shown some putative TCF/LEF binding sites lie in the promoters of *MMP-7*[31], *MT1-MMP*[32], *MMP-2 *and *MMP-9*[12], and via these binding sites, Wnt/β-catenin can directly upregulate these *MMPs*. For instance, it has been found that MMP-7 and MT1-MMP are upregulated by β-catenin/LEF-TCF compound in intestinal tumors [31,32]. Other researches showed Wnt signals can upregulate MMP-2 and MMP-9 expression in T cells and regulate T cell transmigration [12]. In addition, Wnt/β-catenin can also upregulate MMP indirectly. Lowy et al observed MT3-MMP was upregulated in gastric cancer, however, no functional TCF/LEF binding site was found in the promoter of MT3-MMP[30]. Taken together, it is clear that canonical Wnt signal pathway is involved in the upregulation of these MMPs.

Our results did not show MMP-2 and MMP-9 were upregulated by SFRP5 downregualtion, though they were also overexpressed in primary gastric cancers, and can be upregulated in T cells by Wnt signals [12]. And in another study, breast cancers induced by Wnt-1 overexpressed a series of MMPs, including MMP-2 and MMP-9, except MMP-7 [13], a long held Wnt target gene in intestinal tumors. These studies suggest that Wnt target genes may be tissue-specific, though it is true that the regulation of MMP expression is complex and it is certain that Wnt signaling is not alone among numerous factors, such as TNF-α [33], H. pylori [34] in regulating MMP expression in gastric cancer.

It should be noted that downregulation of SFRPs seems to be involved in carcinogenesis via both canonical Wnt pathway and noncanonical Wnt pathway. One recent study has demonstrated SFRP1 can inhibit the canonical Wnt/β-catenin pathway in breast cancer cells[35]. Interestingly, in β-catenin-deficient human mesothelioma cell lines, SFRP4 can still inhibit cell growth and promote apoptosis[36]. Another studies showed noncanonical Wnt pathway is also involved in the regulation of MMPs. For instance, Wnt5a has been found to upregulate MMP1 in endothelial cells[27] and MMP-3 in mouse mammary cells[30]. Therefore up to now, we can not rule out the possibility that SFRP5 downregulation upregulates MMP-7 and MT1-MMP via noncanonical Wnt pathway.

## Conclusion

The results indicate there are frequent occurrences of downregualtion of SFRP5 expression in gastric cancer, primarily due to *SFRP5 *methylation. It seems to be responsible for the upregulation of MMP-7 expression and MT1-MMP expression on the ground that they are inversely correlated with SFRP5 expression.

## Competing interests

The authors declare that they have no competing interests.

## Authors' contributions

ZC designed the study, carried out PCR analysis, analyzed and interpreted the data, and drafted the manuscript. BM performed cell transfection. ZN was engaged in drafting the manuscript and in statistical analysis. WW performed Western analysis. All authors read and approved the final manuscript.

## Pre-publication history

The pre-publication history for this paper can be accessed here:

http://www.biomedcentral.com/1471-2407/9/224/prepub
